# Cognitive virtual microscopy: a cognition-driven visual explorer for histopathology – the MICO ANR TecSan 2010 initiative

**Published:** 2011-01-10

**Authors:** Daniel Racoceanu, Nicolas Loménie, Ludovic Roux

**Affiliations:** 1 French National Center for Scientific Research – IPAL UMI CNRS, Singapore 138632, Republic of Singapore; 2 Department of Computer Science, School of Computing, National University of Singapore, Singapore 117417, Republic of Singapore; 3 University Paris Descartes, 75270 Paris Cedex 06, France; 4 University Joseph Fourier, 38041 Grenoble Cedex 9, France

## 

Within the last decade, histopathology became widely accepted as a powerful exam for diagnosis and prognosis in mainstream diseases such as breast cancer. Currently, analysis of medical images in histopathology largely remains the work of human experts. For pathologists, this consists of hundreds of slides examined daily. Such a tedious manual work is often inconsistent and subjective. The recent cognitive microscope – MICO - ANR TecSan project aims at radically modifying the medical practices by proposing a new cognitive medical imaging environment able to improve reliability of decision-making and prognosis assistance in histopathology. Our goal is to design a generic, open-ended, semantic digital histology platform including a cognitive dimension. MICO combines visual perception, pervasive exploration of whole slide images, context (including uncertainties) modeling, cognitive vision and quality of experience to reinforce a visual diagnosis assistance following an approach centered on the user behavior.

http://ipal.i2r.a-star.edu.sg/project_MICO.htm

